# Promoting Cancer Health Equity: A Qualitative Study of Mentee and Mentor Perspectives of a Training Program for Underrepresented Scholars in Cancer Health Disparities

**DOI:** 10.3390/ijerph19127512

**Published:** 2022-06-19

**Authors:** Anastasia Rogova, Isabel Martinez Leal, Maggie Britton, Shine Chang, Kamisha H. Escoto, Kayce D. Solari Williams, Crystal Roberson, Lorna H. McNeill, Lorraine R. Reitzel

**Affiliations:** 1 Department of Psychological, Health and Learning Sciences, University of Houston, 491 Farish Hall, Houston, TX 77204, USA; arogova@central.uh.edu (A.R.); imarti31@central.uh.edu (I.M.L.); mkbritto@central.uh.edu (M.B.); kdsolari@central.uh.edu (K.D.S.W.); 2 Health Research Institute, University of Houston, 4349 Martin Luther King Boulevard, Houston, TX 77204, USA; 3 Department of Epidemiology, University of Texas MD Anderson Cancer Center, 1155 Pressler St., Houston, TX 77030, USA; shinechang@mdanderson.org; 4 Department of Health Disparities Research, University of Texas MD Anderson Cancer Center, 1400 Pressler St., Houston, TX 77030, USA; khescoto@mdanderson.org (K.H.E.); clroberson@mdanderson.org (C.R.); lmcneill@mdanderson.org (L.H.M.)

**Keywords:** UHAND program, cancer disparities, cancer health equity, women, minorities, mentoring relationships, educational training program

## Abstract

Racial and ethnic minorities, and women, experience stark disparities in cancer risk behaviors and mortality rates, yet often remain underrepresented in scientific research positions. We conducted an exploratory, qualitative study to examine the value of mentored research experience as part of an NCI-funded research training program designed to increase the representation of minority and women scientists in cancer disparities research. Using individual interviews, we explored 16 mentees’ and 7 mentors’ program experiences and perspectives to identify the most effective strategies to build strong mentoring relationships that could ultimately contribute to increased representation in health disparities research. Two expert analysts employed thematic analysis and constant comparison to code, categorize, and summarize the data into themes. Mentees and mentors shared five themes identifying contributions to program success: conditions for building successful mentoring relationships; role of mentor/mentee similarities or differences and their impact on effective collaboration; program elements that fostered developing knowledge, skills, and confidence; program supportive opportunities; and challenges and benefits of in-person vs. virtual program delivery during the COVID-19 pandemic. These findings contribute to improving the quality of training programs for historically excluded trainees to advance their cancer disparities research careers and offer a successful model that can guide similar programs.

## 1. Introduction

There are stark disparities in cancer risk behaviors, incidence, and mortality for racial and ethnic minority (hereafter, minority) groups and women [1,2,3,4,5]. As the proportion of minority individuals within the United States (US) population continues to climb [6], these disparities pose a significant public health crisis. One promising way of addressing these disparities is to train a diverse workforce of cancer disparities researchers, whose demographics (i.e., race, ethnicity, sex, and gender) mirror those most impacted by these disparities. Underrepresentation of minority individuals and women in scientific research positions is not uncommon, especially when looking at more senior academic and scientific positions, such as faculty roles [7,8,9,10]. However, it is critical that minority individuals and women are represented within cancer disparities research teams, in part because they are the very people best positioned to understanding and undertaking the needs of their own communities [11]. This understanding has been met with targeted funding for increasing diversity among faculty [10], as well as for the development of training programs specifically to support the career development of diverse scholars [12,13,14].

One program developed as the result of this funding priority, the UHAND program, was a 1–2-year mentored training program in cancer disparities research that prioritized the recruitment of minority and/or women scholars. The program was developed and implemented within a collaboration between the University of Houston (UH) and The University of Texas MD Anderson Cancer Center (MDACC) [15,16]. The UH has been designated as both a Hispanic-Serving Institution and an Asian American and Native American Pacific Islander-Serving Institution. Furthermore, it is the second most ethnically diverse major research university in the US [17]. The MDACC has been consistently ranked as one of the top two US cancer hospitals every year since 1990, boasting the No. 1 ranking each active year of this training program [16,18]. Thus, the UHAND program was well positioned between these partnering organizations to offer comprehensive cancer disparities training for diverse undergraduate, graduate, and postdoctoral scholars.

The UHAND program was designed by bringing together components of multiple successful training programs. As a result, the program had three components: (1) the Education Program (EP), (2) the Pilot Research Program (PRP), and (3) the Community Outreach Program (COP); each component is described in more detail in Haq and colleagues [15]. The focus of the present study is the EP, though it also touches on the scholars’ experiences with the COP. Through these two components, scholars participated in educational experiences such as cancer disparities seminars, community-based service-learning opportunities, and professional development activities. Most importantly, through the EP, scholars were paired (i.e., in a dyadic (one-on-one) mentoring relationship) with experienced UH or MDACC faculty mentors who were tasked to oversee individual scholar development via their mentored engagement in research projects.

Mentoring relationships are a well-documented strategy for increasing retention of minority and women scholars in scientific fields [19,20,21,22,23,24,25,26]. Mentoring relationships exist ubiquitously across fields, rank, and time. The potential benefits experienced from mentoring relationships, for both the mentor and the mentee, are well-documented [27,28]. For example, mentees report greater intentions to pursue graduate school, increased research productivity (e.g., grant success and publications), as well as greater career success and enhanced personal development [29,30,31,32,33]. Mentors report intellectual stimulation, including being exposed to new ideas and opportunities stemming from mentee interests, as well as professional satisfaction and success [27,34]. Mentoring relationships are also important for mentors because mentorship is sometimes evaluated as a metric of success for promotion, a practice that may become more prevalent over time [35]. However, the existence of a mentoring relationship alone is not sufficient to reap benefits. Instead, both successful and unsuccessful mentoring relationships exist [36]. Despite the potential for gained benefits, there has been surprisingly little research focused on the dynamics that contribute to a successful mentoring relationship [37,38]; what does exist is usually not focused on minority and women scholars [36]. Furthermore, much of the research that examines mentor–mentee relationships focuses almost exclusively on mentees at the undergraduate (e.g., [20,30,39,40,41,42,43,44]) or graduate level [27,45], or draws conclusions about the dyadic relationship without consideration of both the mentee’s and mentor’s perspectives [36]. Finally, there is no single definition that exists for what constitutes a mentor–mentee relationship [37,46]. The literature offers definitions that are more rigid, such as a relationship where the mentor provides “resources (but not obligations), opportunities (but not demands), advice (but not orders), and protection” [47], but also includes more broad definitions, such as “learning that is set up as an apprenticeship model that occurs one-on-one but also in group settings” [48,49].

These gaps in knowledge are concerning for at least four reasons. First, as mentoring relationships continue to be used as a tactic to increase retention of minority and women scholars in scientific fields, the shared characteristics of successful relationships must be identified. Second, minority and women scholars report unique career experiences directly attributable to their sex/gender or race/ethnicity, such as being more likely to experience imposter syndrome [50,51,52,53], discrimination (interpersonal and structural) in the scientific workforce [54,55,56,57,58,59,60], and greater difficulty finding mentors than men [33]. Third, it is vital to understand what contributes to positive mentoring relationships across career ranks, as drop out becomes increasingly likely as minority and women progress in their career [10]. Fourth, mentors report career benefits from mentoring relationships [27,28], highlighting the development of positive mentoring relationships for women and minority mentors as one potential avenue to increase retention; thus, a dyadic approach is warranted.

Most research on the training of underrepresented researchers in biomedical and health-related fields has used quantitative or mixed methods designs [49,61,62,63]. However, more qualitative studies are needed to provide a deeper understanding of underrepresented scholars’ and their mentors’ perspectives and experiences [64,65,66] of the efficacy as well as relevancy of such training programs. The findings presented here qualitatively explored the diverse UHAND mentees’ (undergraduate, graduate, and postdoctoral fellows) and mentors’ program experiences and perspectives to identify effective strategies to increase representation throughout the pipeline and thus diversify this workforce. Past evaluative findings on the UHAND program utilized quantitative data and painted a positive picture of the program, as well as satisfaction with the mentoring relationships across six pre-determined dimensions: maintaining effective communication, aligning expectations, assessing understanding, fostering independence, addressing diversity, and promoting professional development [15]. However, these quantitative findings cannot elaborate on why scholars evaluated the program and their mentors positively. Furthermore, these findings did not include the perspectives of the mentors, only the mentees. By utilizing qualitative analysis, we can ascertain a more dynamic, and dyadic, understanding of participants’ (both mentees’ and mentors’) perspectives of the program to inform the development of future programs and to highlight aspects of positive mentor–mentee relationships for minority and women scholars.

## 2. Materials and Methods

### 2.1. Ethical Approval

The data collected and analyzed for this study were part of the UHAND program. All data collected for the UHAND program were determined to be exempt from Internal Review Board approval by the University of Houston Research Integrity and Oversight Office. As data collection was specifically conducted for purposes of internal program evaluation, this study did not meet the definition of research according to 45 CFR 46.102.

Prior to study participation, investigators (two qualitative experts who had no role in the UHAND program previously) discussed the focus of the study and the nature of the interviews with all mentee and mentor respondents. Given the inherent power differential between mentees and mentors and the small size of the UHAND program, concerns regarding potential identification of participants were discussed. Participants were informed that the information they shared would be kept anonymous, and every attempt was made to minimize loss of privacy, i.e., use of pseudonyms and concealing of identifying particulars. Permission to audio- and/or video-record all interviews was granted prior to participation.

### 2.2. Study Design

We adopted an exploratory, qualitative research design using face-to-face interviews as best suited to addressing our research aim—to better understand mentees’ and mentors’ experiences and perspectives of the UHAND program. A recognized strength of qualitative research methods lies in their ability to capture individuals’ experiences and perspectives [67], providing an “insider” view that facilitates understanding the everyday lives of participants. This study was framed within a social constructionist perspective in which meaning is not inherent in social life but is rather socially constructed by individuals through their experiences, interactions, and understandings of the world [68]. A primary aim of the UHAND program was the reduction of cancer inequities by increasing training of scholars drawn from within the populations that experience these cancer disparities in the US—yet have been historically underrepresented and excluded from training programs and research opportunities focused on addressing the social and health inequities underlying heightened cancer risks. Given this programmatic focus, of particular interest was scholars’ social construction of “social inequality,” and “underrepresentation”; i.e., how they defined, identified with, and perceived the effects of these inequities within their lives and academic experience of the UHAND program [69].

### 2.3. Participants and Setting

As a collaboration between UH, a minority-serving research university, and MDACC, a comprehensive cancer center, this study was conducted within both settings. Participants included former undergraduate and graduate students enrolled at UH, and postdoctoral fellows who were scholars in the UHAND program in cohort 1, a 2-year, in-person program, and cohort 2, a 1-year, online program (due to the pandemic). Mentors in cohorts 1 and 2 were UH or MDACC faculty. We conducted interviews with 16 mentees (2 males) and 7 mentors (1 male). One mentee and 4 mentors failed to participate in interviews. The wide diversity among mentees and mentors is displayed in Table 1 and Table 2, respectively.

While we did not ask our research participants about their gender identity, most of them identified themselves as women and/or female and two identified as men and/or male in the interviews, which corresponds with demographic survey results, according to which 14 mentees self-identified as female (see Table 1). None of our research participants self-identified with any other gender in the interviews. While the terms “female” and “male” refer primarily to biological fact and a sex category, and “woman” and “man” are conventionally used to describe gender, which is a social construction [68], in everyday speech people often use these terms interchangeably [70,71]. We use the term “gender” in our paper rather than “sex,” as we primarily address the social factors that define the unique position of women in academia, including the structural inequalities they experience and the socially constructed roles that often shape their professional experiences.

### 2.4. Data Collection

Total population sampling [73], a purposive sampling strategy, was used in which interviews were sought with the entire population of interest, in this case all program mentees and mentors. Given COVID-19 concerns, all interviews were conducted online via a recorded videoconferencing platform [74]. Interviews were conducted by the first two authors—A.R., a cultural anthropologist, and I.M.L., a cultural anthropologist and public health researcher, both skilled interviewers trained in qualitative research methods—and lasted between 30 and 50 min. Participants were not compensated for participating in interviews.

Separate semi-structured interview guides for mentees and mentors were used to conduct interviews that focused on participants’ experiences of the UHAND program. Data collection took place from June to August 2021. The development of both interview guides was driven by the aims and remained flexible and open to change according to responses in the field [67]. Shared mentee and mentor interview questions included perceptions of the role and responsibilities of a mentor, nature and attributes of successful or unsuccessful mentoring relationships, application or integration of UHAND programmatic elements and mentored research, if and how similarities or differences between mentor and mentee—demographic or otherwise—influenced the mentoring experience and research progress, impact of online vs. in-person program delivery on mentorship relationships and the overall program, and recommendations for program improvements.

Mentees were additionally asked about their motivation for applying to the program, expectations of their mentor and whether these were met, the benefits and skills ensuing from mentorship, mentees’ experience mentoring other students, mentees’ identification and experience regarding being underrepresented and how this affected their UHAND academic experience, impact of the mentoring program on advancing their career path and their development as a researcher, and satisfaction with the program. Additional questions that were only asked of mentors included the types of guidance provided to mentees, quality and degree of mentee preparation for career advancement, experience mentoring UHAND scholars—as similar or different from other mentees considering their underrepresented status—as well as benefits and challenges, and interest in receiving training in mentorship.

### 2.5. Data Analysis

Interviews were audio- and video-recorded and transcribed verbatim using a professional transcription service. Thematic analysis and constant comparison were used to inductively code, categorize, and synthesize data into themes [75]. Two members of the research team, A.R. and I.M.L., both trained in qualitative research, independently coded the first five transcripts. They then met to compare, discuss, and merge their sets of codes to draw up an initial coding frame. Data analysis progressed iteratively using constant comparison, in which emerging data from subsequent data collection were compared within and across previously coded transcripts to condense codes into categories and themes that were drawn directly from the data rather than being determined a priori. The coding frame remained open to allow for the creation and refinement of themes until no new themes emerged from subsequent data collection [67]. Analysts met periodically to discuss the data analysis, reconcile any coding discrepancies, and refine the final coding frame, which was reapplied to all the data. The process of constant comparison also provided analytic rigor by ensuring accurate accounting of all the data, refining and synthesizing of codes, identifying redundancy and appropriateness of categories and themes, and realization of saturation [76]. Atlas.ti 9 (Atlas.ti, Scientific Software Development GmbH version 9.1.7, Berlin, Germany, 2020) was used to manage the data. Pseudonyms are used throughout this article to protect participant privacy and confidentiality.

## 3. Results

As the aim of this study was to evaluate the UHAND program, most questions were designed to reflect on various components of the program with the major focus on mentor–mentee relationships. Themes were largely guided by the researchers’ interests in how the mentored research aspect of the program were experienced by mentees and mentors who participated in the program. Codes and categories were not predetermined and were drawn directly from the data collected from the participants. Analysis of the interview transcripts resulted in five major themes (Table 3): (1) conditions for building successful mentoring relationships; (2) role of mentor/mentee similarities or differences and their impact on effective collaboration; (3) becoming a researcher: the program’s role in developing knowledge, skills, and fostering confidence among scholars underrepresented in the sciences; (4) program supportive opportunities: networking, funded scholarship, peer support, and community service; and (5) challenges and benefits of in-person vs virtual program delivery.

The first four themes were related to mentors’ and mentees’ experience with various aspects of the program and its impact on their professional development and career trajectory. The first theme describes factors, which contribute to successful mentoring relationships. Participants were asked questions about their relationship with their mentors and mentees and what made them more or less successful. The second theme represents potential issues that minority scholars and women might experience in their mentoring relationships and how the dynamics of these relationships is defined by the mentee/mentor demographic and perceived differences and similarities. This theme is particularly relevant to the UHAND program, aimed at increasing the participation of minority individuals and women in cancer disparities research. The third theme represents a combination of reflections by both mentors and mentees on how their participation in this program affected their academic performance, professional development, and career goals. The fourth theme brings together participants’ experiences with other aspects of the UHAND program, which were not directly related to their mentoring experience, but came up in interviews as important elements that facilitated their learning and professional development. The last theme was related to the challenges posed by the COVID-19 pandemic, which disrupted the in-person delivery of the program in its final months for the first cohort of UHAND scholars in 2018–2020 and determined the online mode of program delivery to the second cohort in 2020–2021.

### 3.1. Successful Mentoring Relationship

#### 3.1.1. Effective Communication

Communication was brought up by almost everyone when asked about what made their mentoring relationships successful. Mentees mentioned faculty availability and ability to communicate on a regular basis as an important factor; their ability to listen to the mentee and meet their needs and interests; and being open and honest in their feedback. While mentees emphasized this aspect of effective cooperation more often and focused on it in more detail, mentors also discussed the importance of regular communication and time commitment:

*I think the availability of time is important. Also, the ability to hear what a student has in mind, what their goals are, and what their interests are, and seeing how the research mentor can best support them.*

(Mentee, female, undergraduate, Nisha)

*Time, you have to make time, embed time to make these things happen. It has to be like intentional time, honesty, a little bit of trust. […] I used to make many assumptions about how things should go […] maybe not everybody thinks the same way as you, they don’t value the same things, so kind of provide a space to see what people value here and what is our happy ground. Usually, there’s a happy ground if you’re going to talk about that.*

(Mentor, female, Evelyn)

Scholars who reported not having particularly successful relationships with their mentors referred to the lack of communication as the major reason:

*I think the delayed communication was one of the most major barriers. […] yes, definitely the communication was what made the mentor relationship, I feel like, a negative one.*

(Mentee, female, graduate, Laura)

#### 3.1.2. Scholar Motivation and Mentor Engagement

Mentees discussed the importance of mentors being available, engaged, and taking interest in their scholars and their work. Mentors also emphasized the need to balance their workload to ensure they were able to provide support to their mentees:

*You could be the best researcher in the world and that’s why all students are seeking you but if you only have five on your roster and you only have time for two, well, you’re not a very good mentor for the other three that are left out.*

(Mentor, female, Sheila)

Mentors emphasized a high level of mentees’ motivation and their aligned research interests in cancer research and work with underserved populations as a reason for their successful collaboration. This enabled mentees to get valuable research experience and get well prepared for the next stage of their careers:

*I think the most valuable piece of the program was that I had a student that really wanted to be involved in this line of research and that differs from just the general population of students you get as a professor in the department.*

(Mentor, female, Sheila)

Applicable to all categories discussed above, mentees and mentors underlined the reciprocal character of a successful mentoring relationship, as they discussed how both parties, mentor and mentee, were responsible for completing their goals on time, being responsive, and accountable. Mentees appreciated mentors considering their interests, style of work, and personality, and recognized they needed to consider their mentors’ preferences as well:

*There are always certain things that she does want in a certain way, so adapting to your supervisor is pretty natural in any type of work environment.*

(Mentee, female, graduate, Michelle)

#### 3.1.3. Compassion and Understanding

Mentees often emphasized the personal aspect of relationships, including their mentors being patient, compassionate, and understanding of their personal situation, including conflicting responsibilities mentees had, the learning curve they were going through, and their goals:

*I felt like she was putting me before herself, which was really amazing to feel that she really valued me and wanted to invest in me as an early researcher and as a mentee, that was really nice and important to me. I really appreciated that because not every mentor does that.*

(Mentee, female, undergraduate, Nisha)

Mentees found it important for mentors to be open for discussion, supportive, remain open to their mentees’ ideas, and help them grow, rather than expect their mentees to simply complete pre-defined tasks and assignments; mentors wanted the space to be creative and challenge themselves. While mentors also emphasized the importance of showing understanding and supporting their mentees throughout their training, and adjusting their expectations to respond to the needs of their mentees when needed:

*I think that’s really important just to have somebody to listen to you and not be afraid to tell your ideas […]. If there’s a little give and take, obviously, […] or it can be made better [your idea] that’s something that a mentor should share, but also just helping to grow, help that person grow as an academic.*

(Mentee, female, undergraduate, Camila)

*I think flexibility and patience and understanding and then also, know how to help students raise their expectations for themselves. […] being patient with them as they do develop those skills and I think just being a good listener [Laughter] throughout the process and giving constructive criticism. I think, obviously, we have to give them feedback throughout the entire process but never making them feel like they’re not smart enough or they can’t do it. That’s been a big one for me.*

(Mentor, female, Maria)

#### 3.1.4. Flexibility and Clear Expectations

Having clear expectations and being responsive to the needs of each other was often discussed as a factor contributing to successful cooperation. Some of the things that mentees highly recommended was setting up regular meetings, as they ensured that both mentors and mentees had an opportunity to update each other on task completion, discuss new assignments, ask questions, clarify any misunderstandings, align expectation, and ensure that they remained clear on tasks and expectations. Having a regular meeting with a mentor was described as helping mentees to keep track of the tasks they were assigned to complete, but also to ensure mentors’ accountability and engagement with their mentees’ work:

*I think it was really successful because she took the time to understand how I worked, what I needed, and what I was looking for and what I wanted, and at the same time, I did the same. So, we both went in, knowing each other’s expectations and how we worked and how we could work together.*

(Mentee, female, undergraduate, Nisha)

In the situations of less successful collaborations, lack of clear expectations was identified as a barrier:

*I guess her mentoring style was just ambivalent and maybe a little unpredictable. At times she was more hands-on and at times she wasn’t, so I wasn’t really sure what her expectations were.*

(Mentee, female, graduate, Lina)

### 3.2. Mentor/Mentee Similarities and Differences

We asked mentees and mentors about the importance of similarities, either demographic or perceived, between a mentor and mentee in establishing a successful mentoring relationship. Overall, both mentees and mentors agreed that similarities played a role, but their answers differed in which similarities they found most important.

#### 3.2.1. Gender

Gender similarity was identified by almost every one of the female mentees we interviewed as an important factor. There are two major reasons that female mentees highlighted in discussing why they were more likely to have a successful collaboration with a female mentor: (1) being able to connect better and feeling more comfortable and safer in sharing their ideas and concerns; and (2) being able to learn about specific challenges and experiences of being a woman in academia:

*I, definitely, feel more comfortable with somebody of the same gender as me. Again, it’s that power dynamic that comes into play that makes me feel more comfortable to be around other people that identifies with me.*

(Mentee, female, undergraduate, Camila)

*Just learning from her experiences as a female, how she’s maneuvered it, how being a female has impacted her educational and professional career was insightful, yes.*

(Mentee, female, undergraduate, Nisha)

Having children was mentioned by one of the mentees and one of the mentors as another ground to connect to each other and better understand each other’s needs:

*She has a young child. I have children that are maybe a couple of years older than her child and so at first, we kind of like bonded around, oh, we both have kids, and we would talk about our kids and being moms and stuff […] I had a lot of empathy as a mother.*

(Mentor, female, Jennifer)

Mentors had less focus on gender in their interviews, with some exceptions. One mentor discussed how important it was for her that she was able to support her mentee in overcoming problems that women in academia might encounter:

*[…] being underrepresented for example as a woman in that area, I thought it was important for me to highlight for her what the different options were and that she didn’t have to pigeonhole herself and just take one path […] The really amazing part of that story is that she did perceive with an open mind and look into other programs that she never thought she would ever have a chance in getting into and she got into [graduate program] which, until this day, when I talk to her, she’s like, “I can’t believe I’m here.”*

(Mentor, female, Maria)

#### 3.2.2. Race and Ethnicity

In general, similarities in race and ethnicity were less often identified as an important factor in building successful relationships by both mentees and mentors who agreed that they did not become a decisive factor in building successful mentoring relationships:

*Race is also important to me, but I think, as long as a mentor has an appreciation and understanding for people of other backgrounds, and I guess understands their role and how their bias might come into play, I think as long as there’s an awareness there that it’s okay for the most part.*

(Mentee, female, undergraduate, Camila)

*That [racial and ethnic] difference did not play a role in our relationship whatsoever, in as much as she already has the knowledge that these are disenfranchised groups, and I don’t think her race dictates that knowledge. […] So, I can’t say that our differences in race or ethnicity had an impact on our relationship, our mentor-mentee relationship.*

(Mentor, female, Sheila)

One mentee, who reported that her relationship with her mentor was not particularly successful overall, referred to their shared ethnicity as a ground to connect with her mentor despite other issues they had due to what the mentee saw as insufficient communication and lack of engagement from her mentor:

*I mean, we’re both [race/ethnicity]. […] I feel in the moments that we did have—we could connect with one another is due to those. We’re from the same culture, from some sense, from some sort.*

(Mentee, female, graduate, Lina)

Another mentee, however, shared that she could not establish good relationship with her mentor despite their shared race and/or ethnicity:

*One thing I will say that I was surprised that race didn’t play more of a factor if I’m being honest […] I am surprised about how that wasn’t something that I could use to build rapport enough to get the mentorship that I needed.*

(Mentee, female, graduate, Laura)

However, mentees agreed that while they were not necessarily looking for a person with a similar ethnic and/or racial background as their mentor, it was very important for them to see professionals with similar racial and ethnic backgrounds represented in academia:

*I don’t think I’ve ever seen like a [ethnicity] person in academia or even like [ethnicity] doctor. I think in some ways, just having that representation matters to help you just, I don’t know, believe that what you aspire to do is possible. […] I guess maybe having them as a mentor wouldn’t hurt either but I think if you have a strong mentor that understands your background, whatever difficulties you may be facing, I think it’s the same. At the end of the day, you have a good mentor.*

(Mentee, male, undergraduate, Stephen)

#### 3.2.3. Being Underrepresented

Belonging to a group underrepresented in academia was overall discussed as an important factor to consider in establishing mentoring relationships. In addition to gender, race, and ethnicity, discussed in the above sections, some mentees also considered themselves as being underrepresented in academia based on such factors as being a first-generation American, coming from economically disadvantaged background, or being a first-generation college student. Mentees were particularly appreciative of the opportunity to participate in the program and work with the mentors who were understanding of their backgrounds and had a first-hand experience of some specific difficulties mentees from groups underrepresented in academia face:

*I come from a lower income background and the schools that I attended were underperforming and so there just weren’t a lot of opportunities for things like research, things like science programs and what not until—I think for this program to specifically pick out the people like me to give them a chance to do these types of things is a really good thing. It’s very important.*

(Mentee, male, undergraduate, Patrick)

One of the mentors reflected on how sharing in the position of being a first-generation college student helped her to better understand some of the challenges that her mentee was facing:

*Some of the challenges she encountered, for example, when she was getting ready to apply to graduate school, I recognized in my own challenges, things that we know now that if you’re the first one in your family to go to college or graduate school, you don’t know what you’re looking for or what you should be focused on in the applications. I definitely recognize those challenges in her.*

(Mentor, female, Sheila)

While mentees addressed some of the problems and difficulties that arise from their position of being from groups underrepresented in the sciences, they also identified certain positive aspects. Since the UHAND scholars were engaged in health disparities research with their mentors, they often emphasized how their background helped them to better understand the issues they were studying and people they were working with:

*I guess some of the experiences that I had growing up in lack of access to care, lack of all of those things, growing up low income, single parent or growing up with a single parent, I think all of that it primes me to work with certain populations that experience certain health disparities. There’s a little bit more insight, I think, for sure.*

(Mentee, female, undergraduate, Camila)

#### 3.2.4. Common Values, Interests, and Personality

When we asked mentors and mentees about the importance of having similarities and differences between them, most mentees agreed that similarity played a big role in establishing effective collaboration. However, most of them first talked about the importance of similar interests and values, as well as of a personal connection they could establish with each other. In addition to common professional interests and values, they reported that being able to laugh together, share food, share interest in exercise, and enjoy each other’s company played an important role in shaping effective professional relationships:

*I think the key piece would be similar values. Both of us value similar things, like compassion, diversity, work-life balance; all of those things really helped foster the relationship.*

(Mentee, female, graduate, Shirin)

Mentors discussed how it was difficult to pinpoint exact factors that contributed to their successful collaboration with their UHAND mentees, but they also supported the importance of similar attitudes and interests in addition to their shared minority status or gender:

*We’re both minorities. She can easily see that. […] Although, I don’t think it had played a role in this particular relationship […] my UHAND scholar was just really eager and really ready to learn, and so it just came naturally for the both of us. We just synced, we vibe but, generally speaking, I do believe some of those variants had to do with our similar minority status, although I can’t think of anything that pinpoints it. That’s just my general belief.*

(Mentor, female, Sheila)

*I think we connected a lot on being women and I think just having the same passion for health disparities and just—I mean, a lot of other things. […] the initial thing that connected us when she first started working with me is that we both really enjoyed being physically active*

(Mentor, female, Maria)

One of the mentors further problematized the role that similarities and differences play in establishing relationships between mentors and mentees. She discussed her experience working with one of her mentees, who was very similar to her in a lot of ways and how these similarities shaped her expectations towards this student, which she later had to adjust:

*[I thought] oh, she’s going to do just like me. […] it’s going to be the same, but it wasn’t. […] on the one hand, the similarities create a whole lot of empathy for the student and understanding when they are in situations, but then it can also make you very judgmental about their personal situation, and I think on the flip side, too, when you have differences with a student, like easily observable differences […] I would be trying to be extra sympathetic about things that I couldn’t identify with, [giving] all this extra room for things and flexibility [even when it was not necessarily needed].*

(Mentor, female, Lisa)

### 3.3. Becoming a Researcher

Mentored research experience was designed for trainees to develop the skills and knowledge required to continue their professional careers in the field of cancer prevention and research. During the interviews, we asked participants to reflect on this experience and share their thoughts on the most important outcome of this program for their professional development and career goals. Overall, trainees agreed that the opportunity to work on a mentored research project was an important part of their training. We have identified the following categories in our analysis of the interview transcripts that relate to the theme of professional development.

#### 3.3.1. Building Skills and Knowledge

All mentors and mentees emphasized the importance of the program for developing research skills and knowledge. Mentors had a positive evaluation of research skills mentees gained and/or improved during their time with the program. Mentees discussed the specific skills they learned, including preparing publications, presenting scientific posters, understanding how lab works, etc.:

*[Mentor’s] contributions are huge. Helping me understand, from the basics, how to raise these questions […] and how do you go about redeveloping them if they don’t work, or if what you want to know is impossible? So, in particular working on a paper with the [community partner] and having her guide me in understanding the data, understanding what I need to know, and being able to execute it and her supporting me, and feeling like, I’m going to give this my best shot and she’s there to help me.*

(Mentee, female, graduate, Shirin)

#### 3.3.2. Motivation and Self-Esteem

This category was derived from the interview data as many mentees and some mentors reflected on the role of this program in mentees’ increased self-esteem and confidence in thinking about themselves as “researchers”:

*Just to motivate you as a student to put yourself out there in academia because I think a lot of the undergraduates in my cohort were never in research before and weren’t even sure if they could do research and I think that’s certainly how I felt before UHAND.*

(Mentee, male, undergraduate, Stephen)

*They helped me kind of gaining confidence in myself like to be independent and contribute to the work that they were doing. I definitely have a voice which was nice. […] They helped me become a more independent researcher for sure.*

(Mentee, female, undergraduate, Camila)

Mentees discussed how this increased self-esteem and confidence allowed them to become better professionals and overcome some of the barriers they previously experienced:

*Of course, I can learn how to run a centrifuge and things like that, but I think the biggest thing was the professionalism that I learned working with all these people who are highly esteemed researchers from all over the world […] just being able to talk with them and have a conversation, […] look presentable and be professional.*

(Mentee, female, undergraduate, Tiffany)

Some scholars shared that the participation in the program allowed them to feel support that increased their motivation to build their careers in the sciences:

*Just the ability for, again, Dr. [name] to really just invest in me and put me in front of her sometimes was really helpful, but it also made me see how I want to continue to do that for future generations of researchers. […] now I can also see myself more in academia, to be a mentor and also teach others, but it’s also impacted my career path in the sense that now as I’m progressing through my master’s degree, and hopefully, future MD.*

(Mentee, female, undergraduate, Nisha)

*That motivation is needed, that inspiration is needed in order to get the most—the best quality work from your student […] If the student is not necessarily feeling motivated, or inspired, then that could potentially negatively impact the work that they’re doing with you and the quality of their work. Either because they’re afraid of asking questions or getting direction, or because of just the lack of motivation.*

(Mentee, female, graduate, Laura)

#### 3.3.3. Career Development: Making a Difference

Mentees and mentors were asked questions about mentees’ progress in the program and preparedness for the next stage of their career and professional development. Mentees shared that their mentors played a particularly important role in navigating them through different career opportunities both within and outside of academia:

*I’m definitely well prepared in the sense that I have a better idea of what I want to continue to do and work towards in terms of career goals, but I’m also well prepared in terms of the research skills, experiences, and the career development that I gained from the UHAND program.*

(Mentee, female, undergraduate, Nisha)

Some scholars shared that the participation in the program allowed them to gain confidence in applying for certain jobs and graduate programs:

*She was like motivating me and encouraging me to do it [applying to high-ranking graduate schools]. I think without that motivation and encouragement, I would have kind of been more intimidated by the process, but I was able to get in those schools, I was very happy.*

(Mentee, female, undergraduate, Carmen)

Mentors had overall positive feedback on their mentees’ professional development and preparedness for the next step in their education and professional careers:

*Absolutely successful because I feel like she […] learned what she expected to learn and what I think UHAND expects us to learn out of their research projects, the research part that they engaged in. Also, for me, the biggest part aside from that is that she’s in graduate school now. She’s doing great. She’s successful and she has taken those skills she learned when she was a UHAND scholar working with me and she’s applying them in graduate school. I feel like it was an absolute success.*

(Mentor, female, Maria)

### 3.4. Program Supportive Opportunities and Challenges

The mentored research project experience was a part of the larger education program where scholars were exposed to other learning opportunities. While these were not a focus of our interviews, several themes came up, which were closely related to the mentor–mentee relationship. Funding provided to scholars who participated in the program was mentioned by both mentors and mentees as an important factor structuring their relationship:

*I believe that I got more of a high function of output from her because she had to log hours in. She had to account for the time that she spent on this research.*

(Mentor, female, Sheila)

Mentees discussed the importance of funded fellowship that enabled their participation in the program, which was in particular emphasized by scholars who claimed that they would otherwise not have been able to engage in a long-term research experience as a part of their undergraduate studies:

*I come from a lower income background and the schools that I attended were underperforming and so there just weren’t a lot of opportunities for things like research, things like science programs and what not […] without this program, I probably would have never been introduced to research.*

(Mentee, male, undergraduate, Patrick)

Mentees highly appreciated networking opportunities that they were presented with through this program, in particular through collaboration with MDACC. Some of the mentees were matched with mentors from MDACC, which provided them with opportunities to develop collaborations with faculty working at this institution and expand their learning experiences to a major cancer center.

Another important aspect of the program was peer support that most mentees mentioned as a benefit of the program. This support included other mentees in the program with whom they developed a strong sense of collegiality, but also support from other members of their mentors’ research teams. The importance of other team members was mostly emphasized by students who had less successful relationships with their mentors:

*[…] s/he was a lab manager, and then there was A. who was also his/her mentee and then s/he had another mentee. So, all three of them were a huge help. They were definitely mentors to me. I still keep up with a few of them to this day. So yes, they definitely offered a lot.*

(Mentee, female, undergraduate, Tiffany)

Additionally, the UHAND program gave mentees the opportunity to mentor other students, which was viewed a valuable part of the training program:

*[Mentoring other scholars] exposed me, again to mentorship it’s the same way you’re benefitting from having a mentor. It taught me to also be willing to offer what I knew to my mentees who were undergraduate students and also that prepared me for my faculty role […] working largely with students and also doing research. So I do believe that experience helped a lot with my communication with students at my current position and just preparing me for that role of being a faculty member.*

(Mentee, female, postdoctoral, Alyssa)

### 3.5. Challenges and Benefits of In-Person vs Virtual Program Delivery

The first cohort started the program in person in Fall 2018, but their program was interrupted by the COVID-19 pandemic and the ensuing lockdown that started in March 2020. The first cohort finished their program online, and the second cohort completed a one-year-long program entirely online in 2020–2021. Mentors and mentees were asked about their experience with online and/or virtual participation in the program, and the following categories were derived from the interviews.

#### 3.5.1. Benefits: Accessibility and Affordability

One of the positive aspects of the virtual program delivery was its increased accessibility, primarily in terms of time and opportunity to attend more events and incorporate them more effectively in their schedules:

*It was hard to be remote, and then I realized how many things I’m able to attend, like the programming and the community outreach, and how a lot of it would not be possible if I had to drive from MD Anderson to U of H.*

(Mentee, female, graduate, Shirin)

Some of the mentees also emphasized how this format made the program more accessible to people from underserved groups, not only mentees, but also community members involved in their research projects:

*It made things more feasible for people who also have multiple responsibilities, right? I think that since the grant is working with underserved […] communities and mentees, it’s really helpful to take that perspective […]. So, we all know that people belonging to these groups tend to have multiple life demands and type of roles, and so all of these opportunities might not be feasible.*

(Mentee, female, graduate, Shirin)

*I do think virtual increases equity, especially for those who may have disabilities or dependents at home.*

(Mentee, female, graduate, Lina)

#### 3.5.2. Challenges: Peer Support and Fellowship

A major negative aspect of virtual delivery of the program discussed by mentees and mentors was a limited opportunity for networking and establishing closer relationship with their colleagues. Mentees who attended the program in person assumed that they would not be able to achieve the same level of closeness with other participants in the program or experience the same level of peer support if the program had been virtual. Mentees from the second cohort shared that they were not able to connect to their peers as effectively as they thought they could had they met in person:

*I think the camaraderie I had with my cohorts, we would show up early to things and we would talk and I would get advice from A. […] she always had fantastic things to tell us and we would learn from the undergrads and even still, a year later, a lot of us are still connected and so I think that’s something that would be hard to achieve virtually.*

(Mentee, female, graduate, Lina)

## 4. Discussion

The role of mentoring relationships and opportunities to participate in mentored research projects are significant for the retention of scholars from underrepresented backgrounds in research positions. Mentored research experiences are regarded as a “high-impact practice,” and its benefits are well documented [19,20]. Our study contributes to this body of literature by addressing factors that foster successful mentoring relations between diverse postdoctoral, graduate, and undergraduate scholars and their faculty mentors who participated in an NCI-funded research training program called UHAND [15].

Mentees and mentors who participated in the study consistently cited effective communication as a decisive factor in building a productive mentoring relationship, by which they referred to mentors’ availability to respond to emails, meet with their mentees regularly, and provide feedback in a timely manner. Mentors also emphasized the need to communicate on a regular basis with their mentees to support them in their professional development. However, many mentees also shared in their interviews that it was important for them to feel supported by their mentors. While mentees claimed that they had to adapt to their mentor’s work style and expectations, at the same time, they expected a certain level of flexibility, as well as compassion and understanding from their mentors. Mentors and mentees also shared the importance of being able to connect with each other on a personal level, in addition to sharing common research interests and values. The latter aspect of successful relationships was most often mentioned by mentees, but mentors also reported that they appreciated when they could connect to their mentees on a personal level.

Most of the UHAND scholars we interviewed for this study belonged to one or more groups underrepresented in the field of cancer research, including women scholars, racial and ethnic minorities, first-generation college students, and scholars with disadvantaged backgrounds. Considering this, we asked the scholars about the importance of their mentors being similar to them in some demographic characteristics, including gender, race, and ethnicity. Our findings suggest that gender similarity between mentee and mentor played the most important role for female scholars in this group. Almost every one of our female respondents confirmed that it was easier for them to work with female mentors. The importance of gender in academic mentorship remains understudied [33]. However, there is some prior research that indicated how female scholars and health care professionals might benefit from mentoring by female faculty [77,78,79]. Female mentees might benefit from tailored guidance regarding their work and life balance, timing of maternity leave, and raising children while managing their careers and professional development based on the experience of being a woman and a mother [77]. Other research also found that the mentors’ gender is important for female mentees, as women’s experiences in academia might differ from those their male colleagues’ experience, and female mentors are better equipped to assist their mentees to anticipate and overcome some of the gender-specific barriers [78]. Our findings support and further investigate these claims. The major reason given for this preference of finding a female mentor was a greater personal comfort when sharing their ideas and expressing their thoughts and feelings with a mentor of a similar gender. Mentor–mentee relationships inevitably include a power imbalance, with mentors having control over their mentees work while being in senior positions. These differences are mostly unavoidable, but gender becomes a factor that can potentially mitigate or increase this existing “differential power dynamics” [61]. Gender is also an important factor for establishing connection on a personal level, as one mentor and one mentee specifically reflected on the fact that they were able to better connect better to each other on the grounds of both being mothers. Emotional comfort and ability to better connect is important for a successful mentoring relationship, and our findings show that gender congruence becomes one of the key factors for female mentees to establish an effective partnership with their mentors. Female mentors also shared that it was important for them to assist their female scholars in overcoming some of the barriers that women in academia routinely face when building their careers and striving to achieve work–life balance.

A successful mentored research experience plays an important role in improving skills, but also in building students’ “science identities” [20]. There is little research that specifically examines the process of building science identity, and our study contributes to the limited existing research in the area by addressing how scholars’ participation in mentored research projects contributed to their self-esteem and their self-identification as scientists. An opportunity to participate in mentored research projects has been particularly important for undergraduate students from backgrounds underrepresented in cancer research, as they often face additional challenges to building their confidence and envisioning themselves in the field of research. This program provided them with an opportunity to participate in research projects, ranging from providing assistance with grant applications to conference participation and co-authoring publications. In addition to these research skills, scholars shared how their participation in the program and work with their mentors contributed to the fact that they started thinking about themselves as scientists, envisioning themselves in cancer research. Some of the undergraduate mentees specifically shared that they gained confidence to apply for graduate programs in high-ranking institutions. For this aspect of their professional development, the role of the mentor was particularly important. Mentors also discussed how their own diverse backgrounds, including being first-generation college students, ethnic minorities, and women in academia, helped them to better understand the needs of their scholars, anticipate the barriers they might face, and provide them with tailored advice, training, and motivation.

While mentorship has long been known as a crucial element in professional development and career success for scholars underrepresented in sciences [19,20,21,22,23,24], the UHAND program provided scholars with mentorship opportunities that were also integrated into an education program closely aligned with scholars’ and their mentors’ research focus on cancer disparities. This integration of mentored research experience, professional development seminars, and networking opportunities with cancer researchers at UH and MDACC, as well as community service experience, was overall regarded as successful by most mentees and mentors. The integration of mentorship into a larger program created an opportunity for scholars to develop relationships with their colleagues and allowed them to have a positive program experience even in a few situations when mentoring relationships were not very successful. These scholars reported that they were able to connect to other research staff in the lab, other faculty involved in the UHAND program, as well as their peers who participated in the program. Overall, the education and community service components of the program increased mentees’ motivation to participate in the research projects, and mentors greatly valued the alignment of their own research focus and mentees’ interest in these topics, as well as their level of commitment, which contributed to an overall successful mentored research experience.

The second cohort of the UHAND scholars completed their entire program online due to the COVID-19 pandemic. Prior research asserts that the pandemic had a detrimental effect on mentoring [80]. This format caused certain difficulties for the UHAND program participants as it disrupted the work of some laboratories, limited opportunities to conduct community-based research, and also made networking more difficult. However, several mentees said that the virtual format allowed them to attend more events and made the program overall more accessible to scholars with a lower socioeconomic status, as well as to those who have caregiving responsibilities. With the virtual format, they did not have to drive to different campuses across the Houston area, pay for gas and parking, or find and pay for childcare for the time they would have to attend the same events in person. These findings should be further researched to better understand the impact of virtual learning on scholars from underrepresented groups, but our preliminary data suggest that this “digital divide” has not been entirely negative for this group and the integration of virtual program delivery might be a positive development for minority-serving institutions.

While race and ethnicity can play a role similar to gender in establishing personal connections and reducing power disbalance in mentor–mentee pairs, our findings suggest that racial and ethnic differences were experienced as a lesser factor in contributing to successful mentoring relationships by our respondents. In this finding, we need to consider some limitations of our study, including the fact that all mentees we interviewed were from the same institution, UH. UH is a minority-serving institution, and prior research has indicated that scholars who study and work at minority-serving institutions, as opposed to majority-white institutions, have a better chance to be surrounded by faculty and staff who can serve as their role models and make them feel socially and culturally accepted [81]. Considering this factor, UHAND scholars could be less focused on finding a mentor who would be similar to them in terms of race or ethnicity, as opposed to scholars who attend majority-white institutions. Future research should investigate how attendance at majority-white academic institutions affects minority students’ mentorship experiences as well as their choices concerning mentors. Another limitation of this study is that the extent to which the program’s specific construction or foci as a cancer health equity research program affected the relationships between mentees and mentors is unknown. This was a qualitative study, focused on understanding participants’ experiences rather than causal mechanisms. However, it is likely that these contextual factors influenced the evaluation of the program presented herein, as well as factors such as self-selection into the program. Better understanding the effects of context and research focus on mentor–mentee relationships may be of interest in future work. The sample of scholars and mentors in this study was also primarily female, which limits the transferability [82] of these qualitative research findings to other contexts or settings with other participants. Moreover, longer-term follow-up on scholar’s academic accomplishments and, importantly, resiliency in face of systemic sexism in the academy would be of interest in future work. Additionally, although most mentees and mentors reported favorable experiences of the UHAND program, study participation was based on self-selection. As such, participants’ attitudes may be different from those who chose not to participate. While this paper largely focused on the value of mentorship, future work may also benefit from an examination of factors that can impair mentorship relationships. This information would be especially of value in offering training to mentors in the context of a formal scholar training program such as this one, about how to avoid such mentoring pitfalls. More research is needed on how diverse institutional contexts can impact the implementation and success of similar training programs of minority research scientists. However, a strength of this study is that we report the experiences and perspectives of both program mentees and mentors, thus capturing some of the challenges, benefits, and dynamics of academic mentoring relationships that can inform the development of future mentorship programs focused on diversifying the pipeline in cancer disparities research.

## 5. Conclusions

Mentors and mentees reported that the UHAND program was largely successful and decisive in preparing and ensuring underrepresented scholars’ career advancement as cancer health disparities researchers and professionals. For many scholars, participation in this mentoring program provided them with the opportunities to develop the knowledge, skills, and confidence in themselves to pursue and advance their careers as cancer health disparities researchers and medical professionals, which some originally envisioned as outside their reach given their minority background. This study finding demonstrates the effectiveness of the UHAND program in meeting its goal of training diverse scholars in the cancer health disparities research pipeline. It is precisely the pervasive structural inequities, including inequity in education experienced by racial and ethnic minority scholars across academic levels in the US, that necessitates the development of health career pipeline programs such as UHAND. To this end, our findings contribute to the literature supporting the importance of mentorship to minority and female students’ for developing their “scientific identities”. While mentees acknowledged that racial and ethnic differences can be important factors in mentoring relationships—given the differential power dynamics inherent in mentee–mentor dyads—most reported that shared values and gender played a more crucial role in the development of successful mentoring relationships. Female mentees and mentors attested to the importance of shared gender in establishing successful collaboration. Our findings further confirm that female mentorship is highly valued by female mentees who have reported largely positive experience working with their female mentors. This gender aspect of the mentoring relationship is important to consider when designing training programs to encourage female participation and retention in the profession, as well as fostering a positive image of female role models in academia on different levels of research and career training and mentorship.

Mentors rated their scholars’ research projects highly and they often viewed them as more capable than other mentees they worked with and who were not in the UHAND program. Overall, they considered their UHAND scholars well-prepared for their academic and professional careers. Program participants attest to UHAND having achieved its goal of bolstering scholars’ personal and professional development, providing career guidance to elucidate and align career choice and goals, and equipping scholars with the practical skills, knowledge, and research experience to increase their research productivity. Study findings also attest to the value of mentorship training programs to prepare underrepresented minority and female students for leadership roles in cancer prevention and cancer disparities research. Inclusion of mentees’ and mentors’ experiences of the program was vital to evaluate program effectiveness in meeting scholars’ goals and expectations, to ensure equity, as well as to identify any needed program improvements. Findings from this study can serve to guide the development, implementation, and improvement of other mentorship programs focused on ensuring equal participation, and the success, of underrepresented students in cancer health disparity and health science research.

## Figures and Tables

**Table 1:** Descriptive characteristics of the UHAND scholars ^‡^—Cohorts 1 and 2 **.

Cohort 1					
Scholar	Disadvantaged Background ^†^	First GenerationCollege *	Female Sex	Ethnicity ^††^/Race	University Major
Postdoc	X		X	African Black	Public Health
Grad 1	X		X	African American Black	Psychology
Grad 2		X	X	White	Psychology
Grad 3			X	White	Psychology
UG 1	X	X		African American Black	Health Education
UG 2			X	Asian American	Biology
UG 3			X	Hispanic White	Psychology
UG 4	X		X	African American Black	Public Health
UG 5			X	African American Black and White	Biochemical/Biophysical Science
UG 6	X	X	X	White	Public Health
**Cohort 2**					
**Scholar**	**Disadvantaged** **Background ^†^**	**First Generation** College *	**Female** **Sex**	**Ethnicity ^††^/Race**	**University Major**
Grad 1	X	X	X	African American Black	Health Psychology
Grad 2			X	Asian	Health Psychology
Grad 3			X	Hispanic White	Health Psychology
UG 1	X		X	American Indian/Hispanic	Biology/Health
UG 2			X	Native Hawaiian/Pacific Islander	Public Health
UG 3		X		Asian	Biology/Psychology/Pre-med

Note: ^‡^ This table includes those UHAND scholars that participated in this qualitative study. ^††^ Unless otherwise noted, all ethnicities are non-Hispanic. ** Table 1 is adapted with permission fromRef. [15]. 2021, L. R. Reitzel on behalf of all coauthors. ^†^ Racial and ethnic groups that are underrepresented in health-related sciences, individuals with physical or mental disabilities, individuals from low-income families, and from disadvantaged educational environments [72]; * Scholars who are the first generation in their families to attend college; Postdoc: Postdoctoral Fellow, Grad: Graduate Scholar, UG: Undergraduate Scholar.

**Table 2:** Descriptive characteristics of the UHAND mentors ^‡^—Cohorts 1 and 2.

Mentor Organization	Disadvantaged Background ^†^	First Generation College *	Female Sex	Ethnicity ^††^/Race	Discipline
M 1 UH	X	X	X	White	Counseling Psychology
M 2 MDACC			X	African American Black	Behavioral Science
M 3 UH			X	White	Behavioral Nutrition/Health Psychology
M 4 UH	X		X	White/Hispanic	Health Psychology
M 5 UH			X	African American Black	Community Psychology
M 6 MDACC				White	Experimental Psychology
M 7 UH	X	X	X	White/Hispanic	Health Psychology

Note: ^‡^ This table includes those UHAND mentors that participated in this qualitative study. **^††^** Unless otherwise noted, all ethnicities are non-Hispanic. **^†^** Racial and ethnic groups that are underrepresented in health-related sciences, individuals with physical or mental disabilities, individuals from low-income families, and from disadvantaged educational environments [72]; * Scholars who are the first generation in their families to attend college; M: Mentor; UH: University of Houston, Houston, TX; MDACC: MD Anderson Cancer Center, Houston, TX, USA.

**Table 3:** Themes and categories identified in data analysis of the transcripts of interviews with mentees and mentors.

Themes	Categories
Successful mentoring relationship	- Effective communication - Scholar motivation/mentor - engagement - Compassion and understanding - Flexibility and clear expectations
Mentor/mentee similarities and differences	- Gender - Race/ethnicity - Being first-generation - Interests/values/personalities
Becoming a researcher	- Building skills and knowledge - Motivation and self-esteem - Career development: making a - difference
Program supportive opportunities	- Funded scholarship - Networking and peer support
In-person vs virtual program delivery	- Challenges - Benefits

## Data Availability

The data presented in this study are available on request from the corresponding author. The data are not publicly available due to privacy and confidentiality concerns given the very small group of mentees and mentors and the ability to link the two from the data alone, which could affect dynamics of ongoing mentoring relationships in unknown ways.
